# Cell Nucleus-Targeting Zwitterionic Carbon Dots

**DOI:** 10.1038/srep18807

**Published:** 2015-12-22

**Authors:** Yun Kyung Jung, Eeseul Shin, Byeong-Su Kim

**Affiliations:** 1 Department of Chemistry, Ulsan National Institute of Science and Technology (UNIST), UNIST-gil 50, Ulsan 689-798, Republic of Korea; 2 Department of Energy Engineering, Ulsan National Institute of Science and Technology (UNIST), UNIST-gil 50, Ulsan 689-798, Republic of Korea

## Abstract

An innovative nucleus-targeting zwitterionic carbon dot (CD) vehicle has been developed for anticancer drug delivery and optical monitoring. The zwitterionic functional groups of the CDs introduced by a simple one-step synthesis using β-alanine as a passivating and zwitterionic ligand allow cytoplasmic uptake and subsequent nuclear translocation of the CDs. Moreover, multicolor fluorescence improves the accuracy of the CDs as an optical code. The CD-based drug delivery system constructed by non-covalent grafting of doxorubicin, exhibits superior antitumor efficacy owing to enhanced nuclear delivery *in vitro* and tumor accumulation *in vivo*, resulting in highly effective tumor growth inhibition. Since the zwitterionic CDs are highly biocompatible and effectively translocated into the nucleus, it provides a compelling solution to a multifunctional nanoparticle for substantially enhanced nuclear uptake of drugs and optical monitoring of translocation.

Nucleus-targeting drug delivery systems (DDSs) have attracted significant attention in the biomedical applications, because they substantially increase the healing efficiency especially for tumor therapy^1^,^2^,^3^. Since many anticancer drugs are required to enter the cell nucleus where the drugs damage the genes to stop proliferation of the cancer cell, the construction of the nucleus-targeting DDSs is crucial to treat the tumors.

Significant efforts have been made to develop clinically-used DDS with market approval. In particular, self-assembled polymeric micelles have been extensively used because of their unique advantages such as high loading capacity, preferential accumulation at the tumor site due to enhanced permeability and retention (EPR) effect, and high tunability of chemical and physical characteristics^4^,^5^,^6^,^7^. In spite of their advantages, however, these polymeric carriers require complicated synthetic process and sophisticated delivery strategy as well as needs to be functionalized with the proper ligands such as nuclear localization signalling (NLS) peptides (nuclear membrane-penetrating peptides)^1^,^2^,^3^ on the surface for nucleus targeting. Moreover, since the polymeric carrier itself cannot be acted as a fluorescent imaging probe, it should be conjugated with an organic fluorescent dye for tracking the individual drug delivery event.

As a more promising alternative, recently, semiconductor nanocrystals known as quantum dots (QDs) have been intensively investigated for simultaneous diagnosis and therapy (theranostics)^8^,^9^,^10^. Because the QDs have high resistance to photobleaching, large stokes shift, narrow size dependent emission spectra, broad excitation spectra and long fluorescence lifetime, they can act as an efficient luminescent probe as well as a drug delivery carrier. However, the QDs have also poor colloidal stability in physiological media and nonspecific interaction with biomatter and need to tailor surface functionalities to facilitate the active nuclear entry. Additionally, potential toxicity of heavy metal ions comprising QDs limits their application to a broader scale of clinical setting. Therefore, it remains an important challenge to engineer the nucleus-targeting DDSs with efficient luminescent properties for optical monitoring and capability to reach to the cell nucleus for drug delivery. Herein, we develop a novel nucleus-targeting DDS based on carbon dot (CD) for concurrent nucleus-targeted drug delivery and optical monitoring.

The highly luminescent CDs have emerged as a prospective class of biolabels by virtue of their biocompatibility, low toxicity, and mass production by a simple preparation method^11^,^12^,^13^,^14^,^15^,^16^,^17^,^18^,^19^,^20^,^21^. Furthermore, the CDs contain a sp^2^- and sp^3^-hybridized carbon structure that can load aromatic drugs via strong π–π interactions, making them a promising drug carrier for disease treatment. These properties of the CD allow it to be considered as an effective alternative for nucleus-targeting DDS. However, like many other nanomaterials developed, most CDs reported to date are localized to the cell cytoplasm, including the lysosomes, mitochondria, Golgi apparatus, and endoplasmic reticulum^11^,^12^,^13^,^14^,^15^,^16^,^17^,^18^,^19^,^20^,^21^ (Table S1). Judging by current status and the potential for optical monitoring, it is evident that the next step in the evolution of CD is enabling for it to reach to the cell nucleus without being trapped in the cell cytoplasm. Cytoplasmic and nuclear uptake of nanoparticle cargo in live cells can be determined by the size^22^ and surface charge^23^,^24^,^25^,^26^,^27^,^28^,^29^. In addition, positively charged nanoparticles are preferentially internalized by cells and negatively charged ones interact with nuclei whose pH is consistently 0.3 to 0.5 units above that of the cytosol^23^,^24^,^25^,^26^,^27^,^28^,^29^. Based on this observation, it is expected that the preparation of zwitterionic CDs with both positively and negatively charged functional groups, can facilitate cytoplasmic uptake and subsequent nuclear translocation of theranostic drug-vehicle conjugates. Moreover, it is known that the nanoparticles with zwitterionic surfaces have shown higher colloidal stability over a wide pH range and high resistance to non-specific protein adsorption, thereby prolonging blood circulation for enhanced tumor accumulation^30^,^31^,^32^,^33^,^34^,^35^. Therefore, in this work we have fabricated multifunctional zwitterionic CDs for nucleus-targeting DDS via a simple one-pot synthesis using citric acid (CA) as a carbon source and an amino acid derivative, β-alanine (β-Ala), as a zwitterionic passivating agent, thus avoiding complexity and safety concerns (Fig. 1). *In vitro* study has shown that the synthesized CDs were delivered into cell nuclei by their multicolor fluorescence. Furthermore, the CD-based DDS constructed by the non-covalent grafting of anticancer drug doxorubicin (Dox) not only efficiently accelerated nuclear and tumor accumulation of Dox, but also markedly enhanced the cytotoxicity in cancer both *in vitro* and *in vivo*, which is superior to many other nanoparticle-based Dox delivery systems.

## Results

### Preparation and characterization of zwitterionic CDs

To synthesize zwitterionic CDs with a high quantum yield (QY) by one-step microwave pyrolysis, various ratios of CA and β-Ala were evaluated. As the ratio of β-Ala to CA increased, the peak at 340 nm became broad and increased the QY to 21.9%, as shown by a deeper yellow color under white light and a brighter blue emission under UV irradiation (Fig. S1). Since the QY was saturated at a 1:2 molar ratio of CA to β-Ala, this composition was selected for subsequent studies. In contrast to typical passive dialysis, we purified the as-synthesized CDs by polyacrylamide desalting columns (MWCO 1,800 Da). The fraction with the highest QY was used for the following studies (Fig. S2).

CA/β-Ala CDs showed two characteristic absorption peaks at 248 and 335 nm (Fig. 2a). The peak at 248 nm was related to a typical absorption of an aromatic system, which is suggestive of an sp^2^ carbon network^14^,^15^, and the strong absorption peak at 335 nm was attributed to the n − π* transition of the carbonyl group present on the CD surface from absorption shift of the CD solution as a function of solvent polarity (Fig. S3)^14^,^18^. A bright blue fluorescence under UV light with a maximum emission wavelength at 418 nm was observed upon excitation at 335 nm. The fluorescence emission spectra of CA/β-Ala CDs exhibited an excitation-dependent feature, which is quite different from that of QDs and organic dyes, enabling multicolor fluorescence detection (Fig. S4). It is known that the multicolor photoluminescence of CDs originates from a combination of quantum confinement effects and the distribution of different emissive surface traps^11^,^14^. The exciton lifetime was determined by time-correlated single photon counting (TCSPC), yielding an average value of 4.3 ns, which is suitable for biological applications^19^ (Fig. S5). Bulk production by a simple manufacturing process is another advantage of CDs over other QDs and GQDs, yielding over a gram-scale powder in a highly efficient manner (average isolated yield over 30%, inset in Fig. 2a).

Fourier-transform infrared (FT-IR) spectroscopy was carried out to characterize the chemical functional groups on the CDs. The as-prepared CDs showed peaks at 1174 (C-O stretching), 1772 (C= O stretching), 2947 (C-H stretching), and a broad peak around 3317 cm^−1^ (O-H and N-H stretching) (Fig. 2b). Moreover, the CD spectrum showed two bands at 1708 and 1616 cm^−1^, which correspond to the asymmetric and symmetric stretching vibrations of the carboxylate groups (COO^−^). The successful passivation of β-Ala was confirmed by the presence of amide bond bands at 3095 (N-H stretching) and 1409 cm^−1^ (C-N stretching). In accordance with the FT-IR results, high-resolution X-ray photoelectron spectroscopy (XPS) spectra of the CDs further confirmed the successful surface passivation. For example, the deconvoluted high-resolution spectrum of C1s provided the detailed CD composition, indicating C-C (285.0 eV), C-N (285.7 eV), C-O (286.6 eV), C= O (287.2 eV), and O-C= O (289.0 eV) groups within CDs (Fig. 2c).

The CDs exhibited superior stability in aqueous solution, with a nearly neutral ζ-potential (−5.09 ± 6.47 mV) at pH 7, due to the presence of the zwitterionic β-Ala, which has both negatively charged carboxylic acid and positively charged amine moieties. The surface charge of the CDs gradually increased from −44.1 ± 1.63 to 7.73 ± 1.43 mV with a decrease in pH from 11 to 3 (Fig. 2d). Figure 2e shows a high-resolution transmission electron microscopy (HRTEM) image of the CDs, revealing that the CDs had spherical shape with an average diameter of 3.09 ± 0.51 nm (Fig. S6). Additionally, aberration-corrected HRTEM allows to observe the hexagonal unit cells and a crystalline structure with an interlayer spacing of 0.24 nm (Fig. 2f), which corresponds to (111) lattice spacing of the graphite hexagons as confirmed by the Fast Fourier transform (FFT) profile of a single CD (inset of Fig. 2f)^36^,^37^,^38^. An X-ray diffraction (XRD) pattern with a broad peak was assigned to the interlayer spacing, with a value of 0.478 nm (Fig. S7), which is higher than the values found between the planes of bulk graphite (0.344 nm), suggesting the presence of heteroatoms within the carbon framework^11^,^16^,^18^,^19^.

### Cell nucleus targeting of zwitterionic CDs

In order to attain efficient nuclear drug delivery, it is necessary to evaluate the intracellular uptake and nuclear transport of the CD vehicles. The time-dependent localization of the CDs was studied after incubation with the human cervical cancer cell line, HeLa, for varying lengths of time by monitoring the blue, green, and red multicolor fluorescence signals and the PL spectra of CD itself under ultraviolet (405 nm), blue (473 nm), and green (559 nm) laser excitation taking advantage of multicolor emission of CDs, respectively (Figs 3a and S8) as well as by quantitatively measuring the fluorescence intensity of three circular regions within the cells (Figs 3b and S9). The fluorescence of CDs began to appear in the cytoplasm after 2 h treatment, suggesting that the CDs permeated the cell membrane. After 6 h incubation, the CDs proceeded to move into the nucleus, a partially delocalized fluorescent signal was observed at perinuclear region, and similar levels of fluorescence were monitored on both sides. Noticeably, significantly strong fluorescence of the cells after longer incubation periods (24 h and 48 h) originates mainly from the nuclei. The Bio-TEM images further demonstrated the clear nuclear localization of the CDs, in agreement with the strong fluorescence signals appeared in the nucleus after 24 h incubation (Figs 3c and S10). Whereas the CDs composed of 1:0.5 and 1:1 molar ratio of CA: β-Ala with negative surface charge (−10.2 mV and −8.7 mV, respectively) showed cytoplasmic localization after 24 h incubation in HeLa cells (Fig. S11). It should be noted that, although the CDs were not modified with any nucleus-targeted signals or chemicals, the CDs were successfully internalized into the cells as well as the nucleus which is attributed to the zwitterionic surface state and small size of CDs^22^,^23^,^24^,^25^,^26^,^27^,^28^,^29^,^30^,^31^,^32^,^33^,^34^,^35^. The positively charged surface group on the CDs can effectively interact with the negatively charged cell membranes to enter the cytoplasm^23^ via clathrin- and caveolae-mediated pathway along with the involvement of passive diffusion^24^,^25^. After entering cells, it is speculated that the CDs translocated into endosome are released from endosome due to the charge reversion from the positively charged surface groups to the negatively charged surface moieties on the CDs^26^. It is well known that the particle below 9 nm can freely diffuse into the nucleus through nuclear pore complexes^22^. Additionally, since the nuclear pH is slightly above that of the cytosol, the negatively charged surface moieties on the CDs can interact with the nuclei, aiding nuclear translocation of the CDs^27^,^28^,^29^.

In order to examine which biological processes are involved in nuclear translocation of CDs, we co-incubated the CDs with histones or DNA polymerase in HeLa cells (Fig. S12). Interestingly, co-incubation with histones prevented CD translocation into the nuclei, whereas DNA polymerase did not affect CD nuclear translocation. Histone nuclear import occurs via multiple pathways, including importins, histone chaperones, and nuclear localization signals^39^,^40^,^41^,^42^, whereas DNA polymerase is translocated by nuclear localization signals^43^. Therefore, we hypothesize that the CDs enter the nucleus through the nuclear pore complex due to their small size and surface charges as well as by histone nuclear import-associated process.

To use the CDs as a potential drug delivery vehicle^13^,^15^,^16^,^18^,^21^, the cytotoxicity of the CDs was evaluated in normal human WI-38 cells and cancer HeLa cells by MTT assay. The CDs were incubated for 24 h in a dose-dependent manner (10–1000 μg/mL) in triplicate. As expected, the CDs displayed remarkably low cytotoxicity (Fig. 3d), with greater than 95% cell viability at concentrations up to 500 μg/mL in both cell lines. These results indicate that the CDs are safe as a potential carrier for drug delivery.

### Efficient cancer cell killing by Dox-loaded CD theranostic vehicles

**Figure 1:**
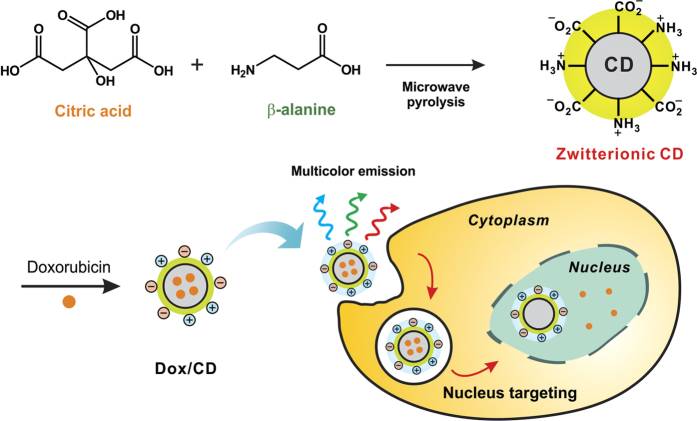
Schematic illustration of the zwitterionic carbon dot (CD) preparation from citric acid (CA) and β-alanine (β-Ala) via microwave-assisted pyrolysis, the fabrication of doxorubicin (Dox)-loaded CD (Dox/CD) and simultaneous cell imaging and efficient Dox delivery to the nucleus by the zwitterionic CD vehicle.

**Figure 2:**
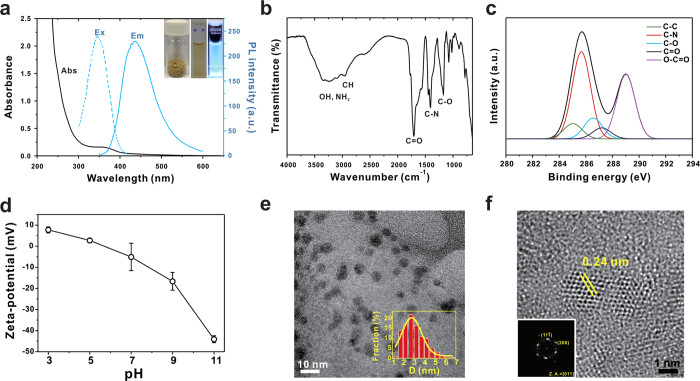
Characterization of zwitterionic CDs. (**a**) UV-vis absorbance, fluorescence excitation (λ_em_ = 435 nm) and emission (λ_ex_ = 340 nm) spectra of the CDs. Inset shows photographs of the CD powder (left), suspension under white light (middle), and suspension under UV light at 365 nm (right). (**b**) FT-IR spectrum and (**c**) deconvoluted high-resolution XPS C1s spectra of CD. (**d**) ζ-potential of CDs with respect to pH. (**e**) TEM image of CDs with a corresponding size distribution histogram. (**f**) High-resolution TEM image showing the arrangement of carbon atoms in CDs with a lattice spacing of 0.24 nm. Inset is the corresponding FFT profile of a CD.

**Figure 3:**
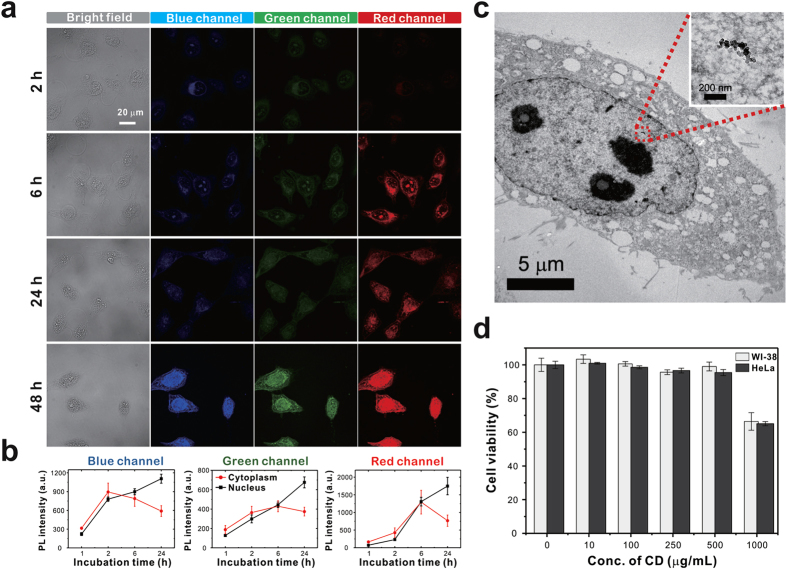
(**a**) Confocal fluorescence microscopy images showing the cytoplasmic and nuclear transport of CDs in HeLa cells. After treatment with 500 μg/mL CDs for varying amount of time, the blue, green, and red fluorescence signals of the CDs were observed under ultraviolet (405 nm), blue (473 nm) and green (559 nm) laser excitation, respectively. (**b**) The fluorescence intensity in the nucleus and cytoplasm is measured separately and plotted in a time-dependent manner for each channel. (**c**) Bio-TEM image of HeLa cells shows nuclear localization of CD. Inset is a zoom-in image of the red box in the main image. (**d**) Cell viability of WI-38 and HeLa cells treated with various CD concentrations for 24 h. The control samples are the untreated cells.

**Figure 4:**
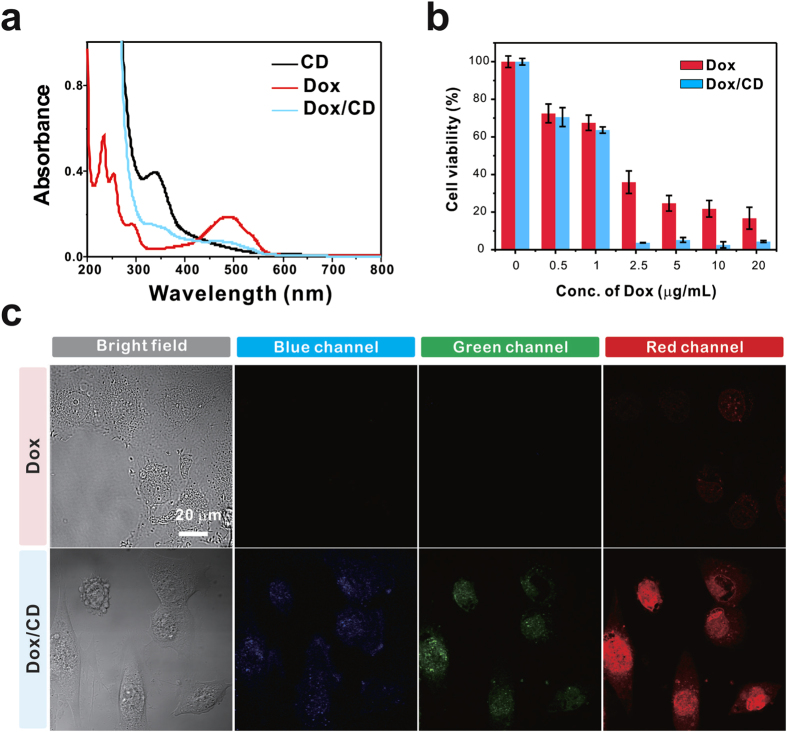
(**a**) UV-vis absorbance spectra of CDs, Dox and Dox-loaded CDs (Dox/CD). (**b**) Cell viability of the HeLa cancer cells exposed to different concentrations of Dox alone and Dox/CD (μg/mL) for 24 h. (**c**) Bright-field and confocal fluorescence images of HeLa cells treated with Dox and Dox/CD (2 μg/mL) for 24 h. Dox/CD delivers Dox to the nucleus more efficiently than Dox alone.

The well-known anticancer chemotherapeutic drug Dox has been widely used to treat a range of cancers, including cervical, gastric, and lung cancer, because it can directly intercalate with DNA to kill cancer cells^44^. However, when Dox is administered directly without a carrier, it displays low anti-tumor activity, due to lack of efficiency in reaching nucleus. The CD-based drug delivery vehicle was constructed by the non-covalent grafting of the anticancer drug Dox via strong π–π staking interactions between the sp^2^-carbon network of CDs and the aromatic structure of Dox (Fig. 1)^11^,^16^,^17^,^18^. The successful loading of Dox onto the CD was evident from the peaks at 339 and 474 nm, arising from respective CD and Dox, resulting in a loading of 14 wt% Dox in the CDs (Fig. 4a). Importantly, the ζ-potential of the Dox/CD complex remained around zero (−5.92 ± 1.20 mV) at pH 7.0, indicating that the zwitterionic properties were preserved.

Next, we examined the anti-cancer efficacy of Dox in HeLa and WI-38 cells. Quantitative MTT assays were carried out by treating both cells with varying Dox or Dox/CD concentrations for 24 h. The HeLa cell viability clearly demonstrated that Dox/CD considerably improved the therapeutic efficacy by 9.7-fold through CD-aided delivery, as compared to free Dox (Fig. 4b). Enhanced Dox uptake by the CD carrier was confirmed by monitoring the red Dox fluorescence (λ_ex/em_ = 480/520–640 nm) under a fluorescence microscope (Fig. 4c). As expected, the cells treated with Dox alone showed weak fluorescence in the red channel, whereas the cells incubated with Dox/CD displayed 4.2-fold more prominent red fluorescence in the nucleus. The fluorescence images are consistent with cancer cell killing by the nuclear accumulation of Dox. It was also confirmed that both Dox and Dox/CD were less cytotoxic to normal WI-38 cells (Fig. S13). Therefore, the Dox/CD complex delivered Dox to the nucleus more efficiently than Dox alone, thereby suppressing cancer cell proliferation and acting as a fluorescent label for intracellular monitoring.

### Enhanced tumor growth inhibition by Dox-loaded CD vehicles *in vivo*

**Figure 5:**
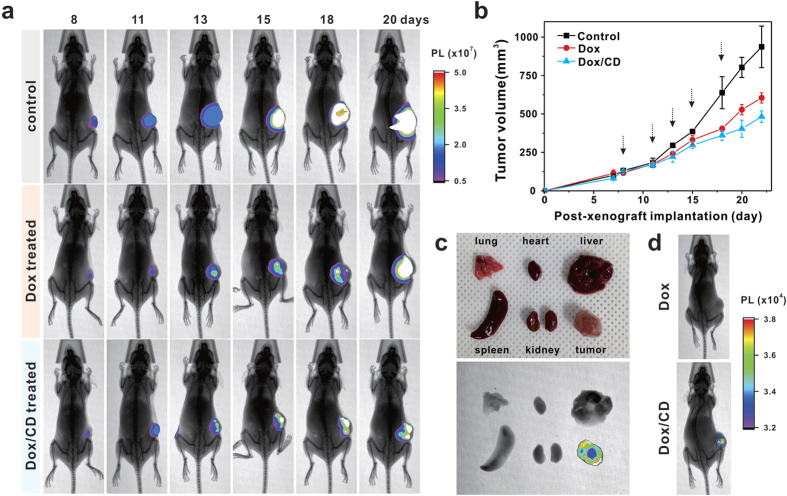
Tumor growth inhibition by Dox/CD in nude mice bearing 4T1-luc2 murine breast cancer xenografts after tail-vein injection of Dox and Dox/CD (*n* = 5, dose: 0.05 mg Dox/kg mouse body weight). (**a**) Luminescence of 4T1-luc2 cells in tumors was imaged after 20 min incubation with D-luciferin. Dox and Dox/CD suspensions were injected into the tail veins of tumor-bearing mice after 8, 11, 13, 15 and 18 days of post-xenograft implantation. (**b**) Volume inhibition (mm^2^) of cancer cell following no treatment (control), free Dox, and Dox/CD treatments. (**c**) *Ex vivo* luminescence images of major organs of mice. (**d**) Luminescence images of tumor-bearing mice after intravenous injection of Dox or Dox/CD. Dox/CD-treated mouse shows luminescent signals on tumors.

The nanoparticles with zwitterionic surfaces have shown higher colloidal stability over a wide pH range and reduced nonspecific interactions with serum components, thereby prolonging blood circulation for enhanced tumor accumulation through the enhanced permeability and retention (EPR) effect^30^,^31^,^32^,^33^,^34^,^35^. We evaluated the accumulation of Dox/CD complexes in tumor tissue after intravenous administration into nude mice bearing 4T1-luc2 breast cancer xenografts (Fig. 5). 4T1-luc2 cells injected into nude mice were used for tumor induction, as well as monitoring of tumor growth by luminescence of cell itself^45^. When the 4T1-luc2 cells expressing luciferase interact with D-luciferin, a luciferase substrate, in the presence of ATP, bioluminescence image can be used to detect progression of tumor growth^45^. To monitor the inhibition of tumor growth by Dox and Dox/CD, each suspension was injected into the tail vein five times at designated time points, and luminescence images of the whole body were collected. Fig. 5a showed that the mouse with no treatment had fast-growing luminescence signals corresponding to rapid increase in tumor size over time. In contrast, the mice treated with Dox or Dox/CD exhibited moderate or slow increases in luminescence signals. This discrepancy was confirmed by changes in tumor volume (Fig. 5b). The tumor inhibition effect of Dox/CD and free Dox treatment was quantitatively analysed by comparing with tumor volume of no treatment (control) object, displaying 48 and 35% inhibition of tumor growth, respectively, after 22 days. Based on the results of the *in vivo* test, inhibition of cancer cell growth was improved by 13% with the Dox/CD complex compared to free Dox. *Ex vivo* luminescence was further examined by imaging major organs that were excised after sacrificing the mice administered with the luciferin. Interestingly, the luminescence was detected only in the tumor tissue, suggesting enhanced EPR effect of Dox/CD (Fig. 5c). Together with the *in vitro* nucleus-targeting capability of CD, the *in vivo* mice assay revealed an enhanced anticancer effect using Dox/CD, as compared to free Dox.

In a separate study, tumor-bearing mice without D-luciferin treatment were intravenously injected with Dox or Dox/CD suspensions after 22 days of post-xenograft implantation (Fig. 5d). Dox-treated mouse did not show any signal, however, Dox/CD-injected mouse displayed its luminescent signal on the tumor. These results indicate that our CDs can emit their photoluminescent signals even *in vivo*. Thus, the Dox-loaded CD delivery vehicle can effectively enhance the accumulation of Dox in tumors via its zwitterionic properties, consequently leading to an enhanced anticancer effect.

## Conclusions

In summary, we report the development of a multifunctional zwitterionic CD that combines imaging, nucleus targeting, and *in vivo* therapeutic efficacy into a single carrier. The use of zwitterionic CDs is not limited to optical monitoring, but also can be used to deliver drugs to the cell nucleus and to tumors. The CDs have excitation-dependent tunable photoluminescence, enabling optical monitoring of the cells in blue, green, and red fluorescence channels, which is not typically possible with organic dyes or quantum dots. These characteristics improve the accuracy of the CDs as an optical code both *in vitro* and *in vivo*. The biocompatible CDs are effectively transported from the cytosol to the nucleus by virtue of their zwitterionic surface charge and small size. Additionally, nuclear import of the CDs is found to be related to the histone transport pathway. Following nuclear translocations, the CDs are engineered to deliver Dox that targets the nucleus. The Dox/CD conjugates considerably improve therapeutic efficacy by 9.7-fold in cancer cells by 4.2-fold enhanced nuclear transport of Dox, and inhibit tumor growth by prolonging the accumulation of Dox/CD in tumour tissues, as compared to free Dox. Therefore, the zwitterionic CDs with good biocompatibility, low cytotoxicity, excellent solubility, and stable photoluminescence are a promising multifunctional platform capable of expediting and sensing the delivery of drugs in a simple and efficient manner.

## Methods

### Materials

Citric acid, β-Ala, Dox, and thiazolyl blue tetrazolium bromide (MTT) assay kit were purchased from Sigma-Aldrich (St. Louis, MO, USA). 10× phosphate buffered saline (PBS), Dulbecco’s Modified Eagle’s Medium (DMEM), fetal bovine serum (FBS), Roswell Park Memorial Institute (RPMI) 1640 media, sodium bicarbonate, penicillin-streptomycin, and trypsin-ethylenediaminetetraacetic acid (EDTA) were purchased from Life Technologies. Polyacrylamide 1800 Desalting Gel and Nunc Lab-Tek II Chamber slides were obtained from Thermo Scientific (Rockford, IL, USA). HeLa (human breast carcinoma cell) and WI-38 (human lung normal cell) lines were obtained from the Korea Cell Line Bank (Seoul, Korea). 4T1-luc2 murine breast cancer cell was obtained from lab of Professor Suh, Pann-Ghill of UNIST, Korea. All solvents used in this study were of analytical grade.

### Preparation of zwitterionic CDs

CDs were synthesized by a commercial microwave (700 W). Firstly, 1.0 g (5.2 mmol) of citric acid was diluted with 10 mL of distilled water and mixed with different amount (2.6 mmol, 5.2 mmol, 10.4 mmol, 15.6 mmol, and 20.8 mmol) of β-Ala. Then, the transparent solution was placed into a microwave oven and heated for 3 min to proceed carbonization and surface passivation. After cooling down to room temperature, the obtained yellow-brown solid was dissolved into distilled water and filtered with a syringe filter (0.45 μm) to remove salt and unreacted residues. Finally, the solution was filtered against DI water through a polyacrylamide desalting columns (MWCO: 1,800 Da) (Thermo Fisher Scientific Inc., PA, USA), collecting the same volume of solution that emerged from the column. To observe the quantum yield (QY, %) according to the ratio between citric acid and β-Ala, quinine sulfate in 0.1 N sulfuric acid solution was used as a reference at excitation wavelength of 360 nm.

### Characterization of CDs

UV/vis spectrophotometer (UV-2550, Shimadzu) was used to record absorbance of CD and Dox-loading CD. Fluorescence data was obtained by using a fluorometer (Cary Eclipse, Varian, UK). A maximum excitation peak at 345 nm was exhibited by 418 nm emission. High resolution-transmission electron microscopy (HR-TEM, JEM-2100F, JEOL) analysis was performed to investigate the size and morphology of the CDs. To confirm the functional groups of CD, FT-IR (670-IR, Varian, UK) and high-resolution X-ray photoelectron spectroscopy (XPS) analyses (K-alpha, Thermo Fisher) were performed. XPS confirmed the successful surface passivation, showing that the CDs contain carbon (C 1s, 286.34 eV, 62.73%), nitrogen (N 1s, 401.14 eV, 8.46%), and oxygen (O 1s, 533.14 eV, 26.64%). The X-ray diffraction (XRD) pattern with a typical peak at 2θ = 19.7° was obtained on a Rigaku D/Max-2500 diffractometer (Japan) to investigate the structural characteristics of the CDs. Zeta potential was measured using a Malvern Zetasizer Nano-series (ZEN3600, Malvern, UK) to characterize zwitterionic properties of CDs.

### Fabrication of Dox-loaded CD (Dox/CD)

Identical volume of Dox (0.1 mg/mL) and CD (1 mg/mL) aqueous solutions was mixed sufficiently and incubated for overnight in dark. The mixture was purified with molecular weight cut-off Microcon (3,000 Da, Millipore Cor., MA, USA). The UV-vis absorption of CD, Dox, and Dox/CD complex was then recorded. By using the absorbance data depending on the concentration of Dox alone as a reference, the concentration of Dox in Dox/CD complex was calculated.

### Cell imaging

Fluorescence imaging of cells was obtained using laser confocal scanning fluorescence microscopy (LCSM, FV1000 SPD). HeLa cell was seeded into each well of an eight-chamber slide at a density of 2 × 10^4^ cells per well and incubated for 24 h in 5% CO_2_ at 37 °C. After removing the culture medium, the wells were washed with 1× PBS. Each well was then replaced with 180 μL of fresh medium and 20 μL of CDs solution composed of 1:0.5, 1:1, and 1:2 molar ratio of CA: β-Ala. After 500 μg/mL of CDs were treated for 2 h, 6 h, 24 h, and 48 h, blue, green, and red fluorescence signals of CDs were observed with a confocal laser scanning microscope (Zeiss LSM 510 META, Jena, Germany) under ultraviolet (405 nm), blue (473 nm), and green (559 nm) laser excitation with 1000× magnification, respectively. The quantitative analysis of fluorescence intensities in triplicate regions of three cells were processed with the aid of MetaMorph software.

### *In vivo* anticancer assay of Dox/CD conjugates

The study in mice was performed in accordance with the approved institutional guidelines and regulations, and all experimental protocol were approved by the Institutional Animal Care and Use Committees (IACUC) of the Ulsan National Institue of Science and Technology (approval number: UNISTIACUC-14-026). Female BALB/c nude mice (6 weeks old, 20 g) were purchased from OrientBio (Sungnam, Korea). Tumor-bearing mice were prepared by subcutaneously injecting a suspension of the 4T1-luc2 cells (3.38 × 10^6^ cells) in sterilized 1× PBS (*n* = 6). 4T1-luc2 cells expressing luciferase were used for tumor induction and detection of bioluminescence signals which reflect tumor growth after addition of D-luciferin. When the tumor size reached around 100 mm^3^, Dox and Dox/CD in 1× PBS solution (0.02 mg/mL, 50 μL) were injected into the tail veins of the tumor-bearing mice. As a control, one group of mice was treated with the same volume of 1× PBS. Luminescent signals from the mice were obtained by using an optical molecular imaging system, *in vivo* xtreme 4 MP (Bruker, USA), at various time points and analyzed by using Bruker molecular imaging software. To examine the ex vivo luminescence images, major organs (lung, heart, liver, spleen, kidney, and tumor) were collected into a petri-dish and imaged. Tumor-bearing mice after intravenous injection of Dox or Dox/CD were imaged after 20 min incubation to observe luminescence signal of Dox or CD itself *in vivo.* The therapeutic effects by Dox or Dox/CD were investigated by monitoring the change in tumor volumes and body weight in each group every two days. The tumor volumes were calculated by using the equation of π/6 × length × width × height, where the length and width are the longest and shortest diameters (mm) of the tumor, respectively.

## Additional Information

**How to cite this article**: Jung, Y. K. *et al.* Cell Nucleus-Targeting Zwitterionic Carbon Dots. *Sci. Rep.* **5**, 18807; doi: 10.1038/srep18807 (2015).

## Supplementary Material

Supplementary Information
