# Supplementary material for: Cell Nucleus-Targeting Zwitterionic Carbon Dots
Source: Sci Rep. 2015 Dec 22;5:18807. doi: 10.1038/srep18807 (PMC4686939; doi:10.1038/srep18807)
Supplement: Supplementary Information [file srep18807-s1.pdf]

Cell Nucleus-Targeting Zwitterionic Carbon Dots

Yun Kyung Jung[1], *Eeseul Shin*[1], *and Byeong-Su Kim*[1,2,*]

[1]Department of Chemistry and [2]Department of Energy Engineering, Ulsan National Institute of Science and Technology (UNIST), UNIST-gil 50, Ulsan 689-798, Republic of Korea

E-mail: bskim19@unist.ac.kr

Determination of quantum yield (QY). The QY of the CDs was calculated by comparing the integrated fluorescence intensity (excited at 360 nm) and absorbance at 360 nm with those of the reference quinine sulfate (QS) [S1-2]. The QS (QY = 0.54 at 360 nm) was dissolved in 0.1 M H_2SO_4 (refractive index (η) of 1.33) and the CDs were dissolved in distilled water (η = 1.33). To prevent re-absorption, CD and QS solutions were diluted such that the absorbance at the excitation wavelength was below 0.1. The QY of the CDs was calculated according to the following equation:

$$QY_{CD} = QY_{QS} \times \frac{I_{CD}}{I_{QS}} \times \frac{A_{QS}}{A_{CD}} \times \frac{\eta_{CD}^{2}}{\eta_{QS}^{2}}$$

where QY is the quantum yield, I is the measured integrated emission intensity, A is the optical density, and η is the refractive index, respectively.

Absorbance measurement of the CD solution as a function of solvent polarity. To examine that the 335 nm peak indicates n - π*, absorbance of the CD solution was measured

depending on polarity. Four different solvents (ethanol (EtOH), dimethyl sulfoxide (DMSO), dimethylformamide (DMF), and tetrahydrofuran (THF)) were mixed with distilled water and the CDs, producing their final concentration of 50%, 83%, and 91% (vol/vol).

Photoluminescence lifetime measurement. The exciton lifetime was determined by the time-correlated single photon counting (TCSPC) technique. The computer controlled diode laser with 375 nm wavelength, 54 ps pulse width and 40 MHz repetition rate was used as an excitation source. The PL emission was spectrally resolved by using some collection optics and a monochromator (PicoQuant). The TCSPC module (PicoHarp 300E, PicoQuant) with a MCP-PMT (R3809U-5x series, Hamamatsu) was used for ultrafast detection. The total instrument response function (IRF) for PL decay was less than 30 ps, and the temporal time resolution was less than 10 ps. The deconvolution of actual fluorescence decay and IRF was performed by using a fitting software (FlouFit, PicoQuant) to deduce the time constant associated with each exponential decay.

Cell culture. HeLa cells, derived from human epithelial carcinoma cells, were incubated with Dulbecco's Modified Eagle's Medium (DMEM, Life technologies) with 10% fetal bovine serum and 1% penicillin-streptomycin. WI-38 cells, derived from human diploid cells, were incubated with Roswell Park Memorial Institute (RPMI) 1640 media (Life Technologies) with 10% fetal bovine serum, 25 mM sodium bicarbonate and 1% penicillin-streptomycin.

Bio-TEM. The HeLa cells were incubated with 500 μg/mL of CDs for 24 h. Then, the cells were washed twice with 1 × PBS. The HeLa cells were fixed by glutaraldehyde at room temperature, then rinsed with PB and dehydrated through a graded ethanol series, finally

cleared with propylene oxide. Then, the cell sample was embedded in EPOM812 and polymerized in the oven at 37 ℃ for 12 h, at 45 ℃ for 12 h and at 60 ℃ for 48 h. Ultrathin sections of approximately 70 nm thick were cut with a diamond knife on a Leica UC6 ultramicrotome and transferred to the copper grid. The sample was stained with uranyl acetate for 10 min and with lead citrate for 5 min. The images were viewed on JEM-1230 electron microscopy.

Co-incubation of the CDs with histones or DNA polymerase in HeLa cells. HeLa cell was seeded into each well of an eight-chamber slide at a density of 2×10^4 cells per well and incubated for 24 h in 5% CO_2 at 37 ℃. After removing the culture medium, the wells were washed with 1 × PBS. Each well was then replaced with 175 μL of fresh medium, 20 μL of CDs solution, and 5 μL of histone H2A (1.0 mg/mL, New England BioLabs® Inc., UK) or 5 μL of DNA polymerase (5 units/μL, Bioneer, Korea). After 24 h incubation, blue, green, and red fluorescence signals of CDs were observed with a confocal laser scanning microscope (Zeiss LSM 510 META, Jena, Germany) under ultraviolet (405 nm), blue (473 nm), and green (559 nm) laser excitation with 1000× magnification, respectively.

Cytotoxicity test of CDs, Dox, and Dox/CD conjugates. HeLa and WI-38 (human diploid cells) were purchased from the Korean Cell Line Bank (Seoul, Korea). Cell viability was assessed by the MTT assay (Sigma-Aldrich). Cells were seeded in 96-well plates at a density of 1×10^4 cells per well and incubated for 24 h in 5% CO_2 at 37 ℃. After removing the culture medium, the wells were washed with 1 × PBS. Each well was then replaced with 90 μL of fresh medium and 10 μL of 10 × CDs solution. After 24 h in 5% CO_2 at 37 ℃, MTT agent was added to each well of cells (final concentration: 0.50 mg/mL) and incubated for 4 h

in an incubator. 100 μL of DMSO was added to solubilize the MTT-formazan product and the sample was incubated for further 15 min at room temperature. Absorbance of the solution was read at a test wavelength of 540 nm.

References

S1. Zhu, H. *et al*. Microwave synthesis of fluorescent carbon nanoparticles with electrochemiluminescence properties. *Chem. Commun.* 5118-5120 (2009).

S2. Zhu, L. *et al*. Fluorescence immunoassay based on carbon dots as labels for the detection of human immunoglobulin G. *Anal. Methods* **6**, 4430-4436 (2014).

S3. Hu, M., Tian, F., Zhao, Z., Huang, Q., Xu, B., Wang, L.-M., Wang, H.-T., Tian, Y., He, J. Exotic Cubic Carbon Allotropes. *J. Phys. Chem. C* **116,** 24233-24238 (2012).

Table S1. Reference papers with cellular location of CDs depending on surface charge.

	Material source	Method	Surface charge (mV)	Cellular location	Reference
**1**	Citric acid, HPAA	Hydrothermal	-30.7	Cytoplasm & nucleus	J. Mater. Chem. B **2015**, 3, 700
**2**	Soot	Nitric acid oxidation	-31.92	Cytoplasm	Adv. Mater. **2012**, 24, 5104
**3**	Ethylenediamine	Microwave	+0.12	Cytoplasm	Chem. Commun. **2013**, 49, 403
**4**	Used green tea	Autoclave calcination	-17.2	Cytoplasm	J. Mater. Chem. B **2013**, 1, 1774
**5**	Nanodiamond	Hydrothermal	-32.7	Cytoplasm	J. Colloid Interf. Sci. **2013**, 397, 39
**6**	Glucose, Leucine	Microwave	0.23	Cytoplasm	Sci. Rep. **2014**, 4, 3564
**7**	Formaldehyde	Hydrothermal	-0.025~-0.078	Cytoplasm	Nanoscale **2014**, 6, 9071
**8**	Branched PEI	Oxidation & Hydrothermal	+23.8	Cytoplasm	Carbon **2014**, 67, 508
**9**	Streptomycin	Hydrothermal	-29.4	Cytoplasm	Analyst **2014**, 139, 1692
**10**	Boric acid, Ethylenediamine	Hydrothermal	-25	Cytoplasm	J. Mater. Chem. C **2015**, 3, 6668

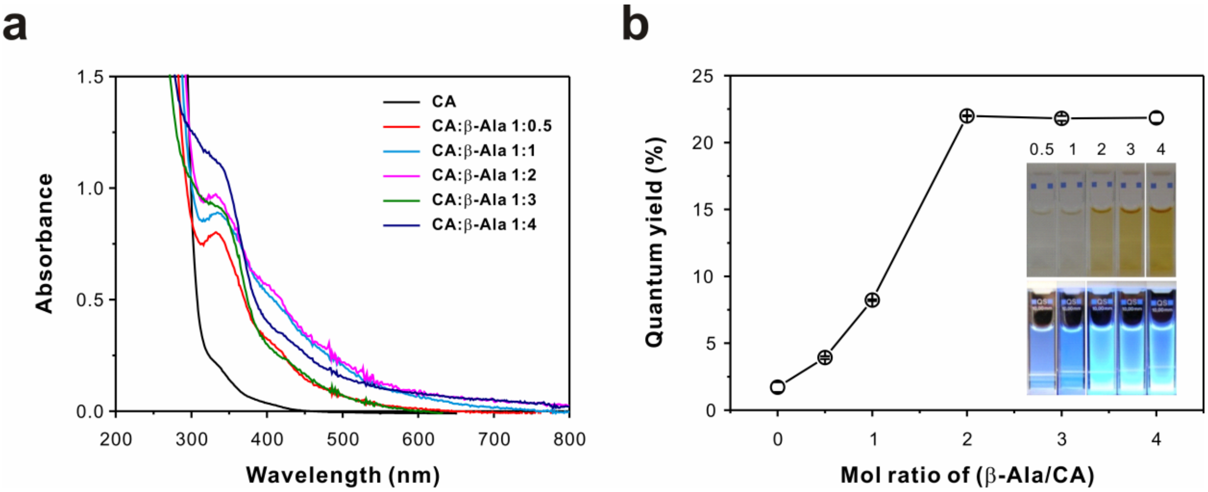

Figure S1. (a) UV-vis absorbance spectra and (b) quantum yield (QY, %) of the CDs depending on the ratio between citric acid (CA) and β-alanine (β-Ala). The inset of (b) shows photographs of the CD solutions under daylight and UV light (365 nm). QY (%) is saturated when the CD is composed of 1:2 molar ratio of CA:β-Ala.

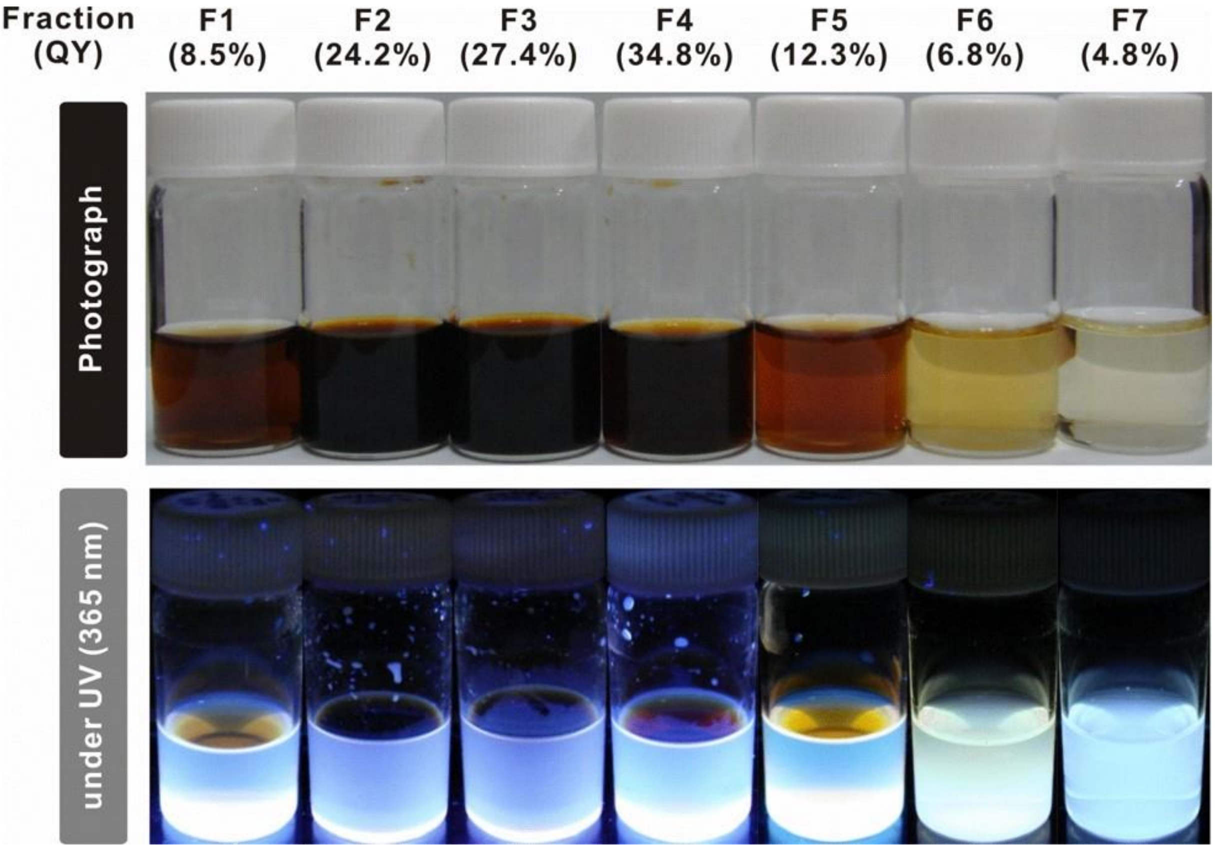

Figure S2. Change in the QY (%) of fractions (F1 - F7; 5 mL each) during column purification of CDs composed with CA:β-Ala (1:2 molar ratio). The photographs show each fraction under white light and UV light (365 nm).

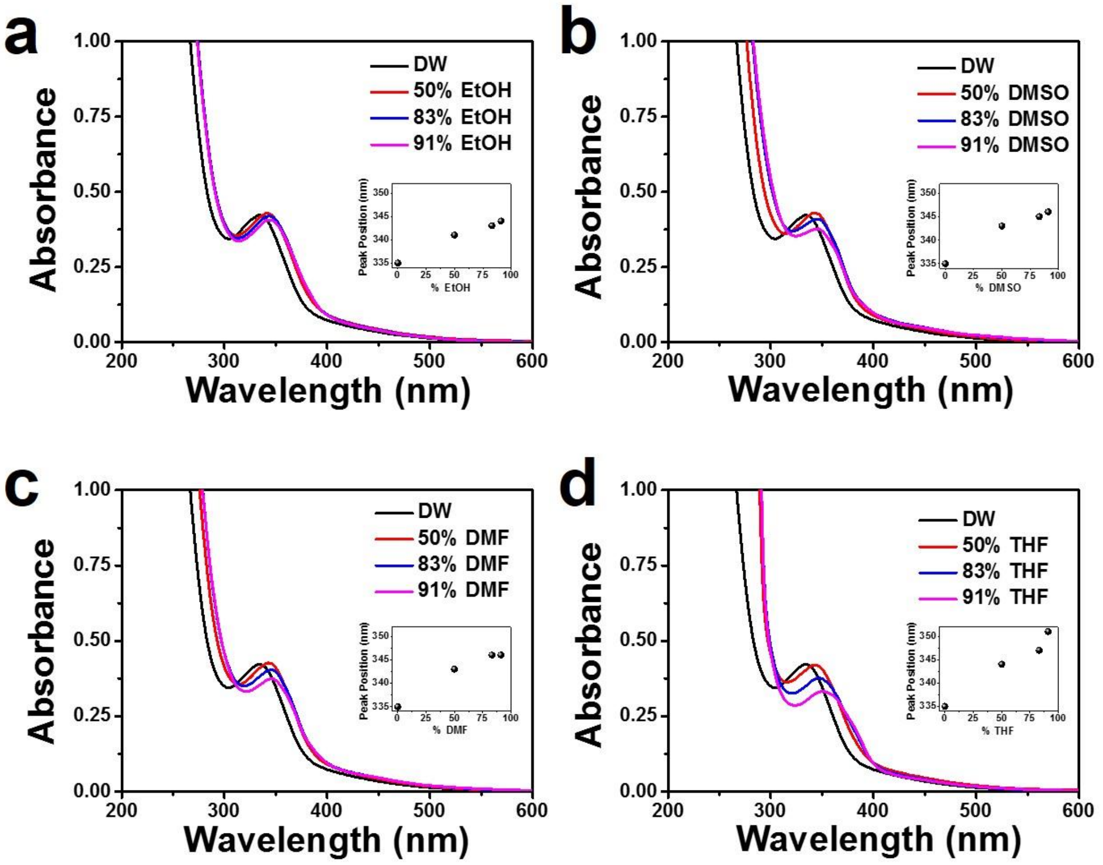

Figure S3. Absorption shift of the CD solution as a function of solvent polarity.

The CD solution dissolved in distilled water has an absorption peak at 335 nm. As the concentration of EtOH increases, the peak position is red shifted (ΔAbsorbance (A)$_{EtOH}$ = 6, 8, and 9 nm). An increase in the amount of DMSO also leads to a red shift (ΔA_{DMSO} = 8, 10, and 11 nm). And, in case of DMF and THF addition, a red shift was also observed with decreasing polarity (ΔA_{DMF} =8, 11, and 11 nm and ΔA_{THF} = 9, 12, and 16 nm, respectively).

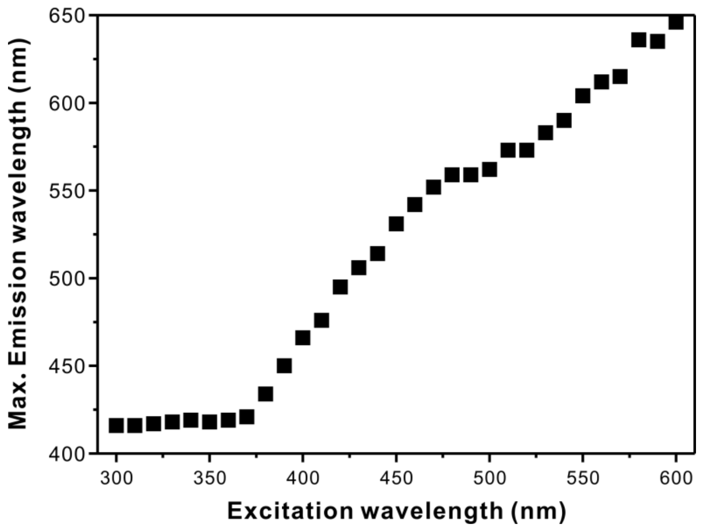

Figure S4. Changes in maximum emission wavelength depending on the excitation wavelength of CD with 10 nm increments.

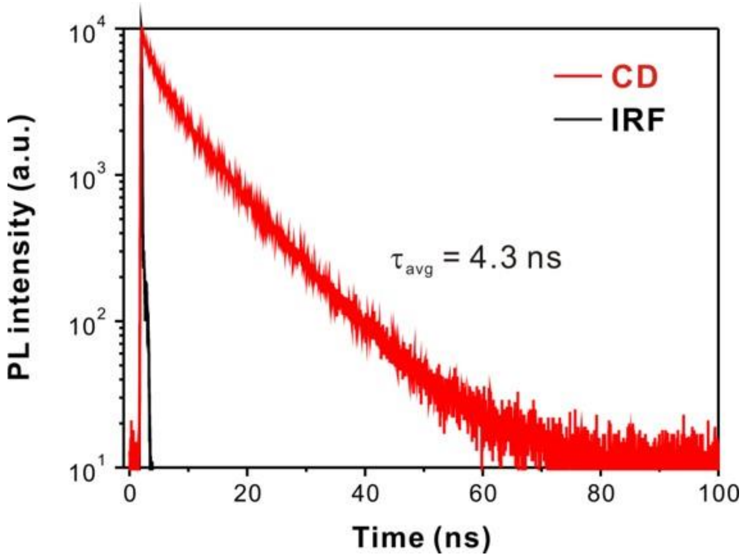

Figure S5. Time-resolved photoluminescence decay curve measured using time-correlated single photon counting (TCSPC) and the average exciton lifetime (τ_{avg}) of zwitterionic CD.

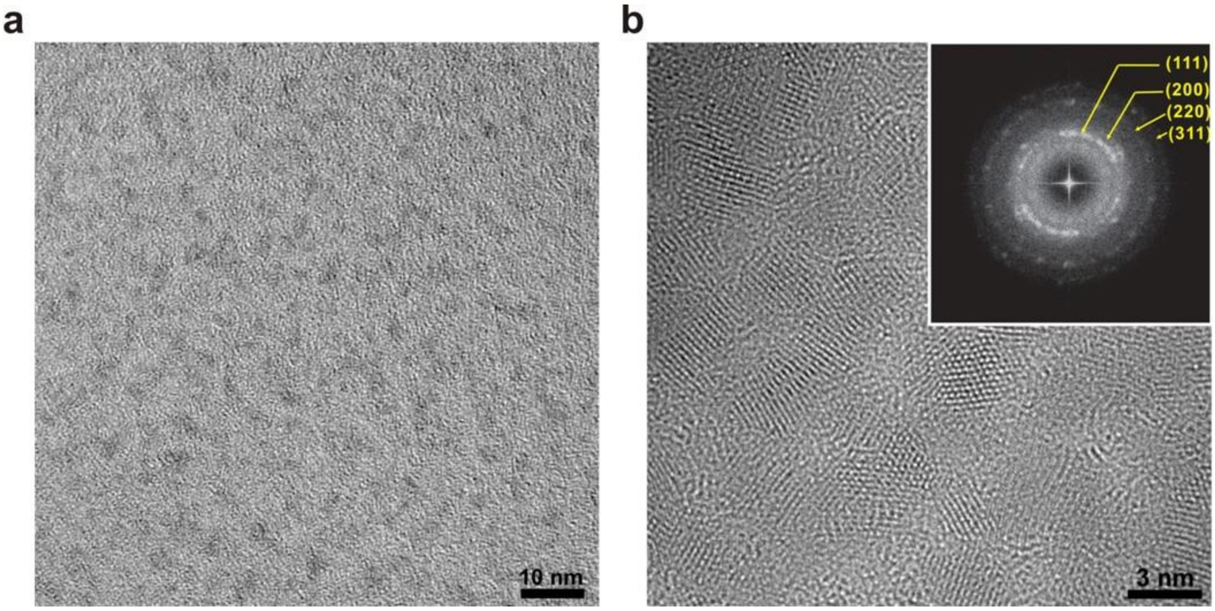

Figure S6. HRTEM images of the CDs at different magnifications (a) 130,000× and (b) 340,000×. Inset in (b) shows the corresponding Fast Fourier Transform (FFT) profile of several CDs, which is equivalent to an electron diffraction pattern. The FFT pattern shows that the CDs possess a face centred cubic (fcc) structure with a lattice constant of a = 4.2 Å [S3].

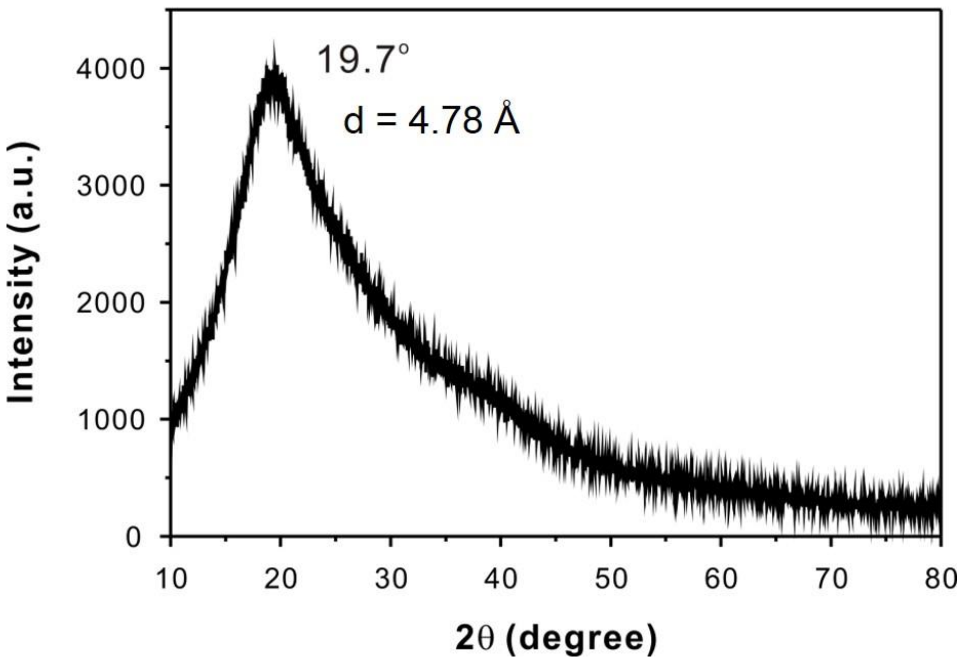

Figure S7. X-ray diffraction (XRD) pattern of zwitterionic CD.

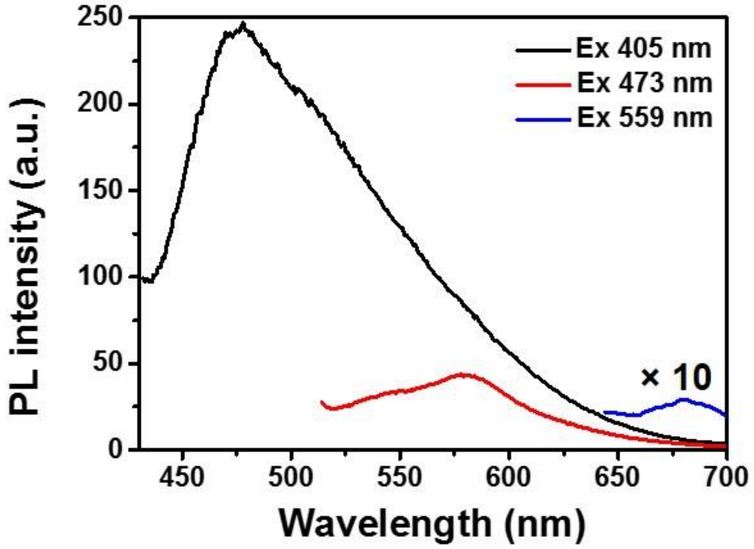

Figure S8. The emission spectra of the CDs depending on excitation wavelength used for cell imaging. With the increase of the excitation wavelength from 405 to 559 nm, emission peaks are red-shifted, while the PL intensities are decreased. The maximum emission intensity upon 405 nm excitation is 5.7-folds and 81.8-folds higher than those upon excitation at 473 nm and 559 nm, respectively. The full width at a half maximum (FWHM) for excitation at 405 nm, 473 nm, and 559 nm is 107 nm, 92 nm, and 26 nm, respectively.

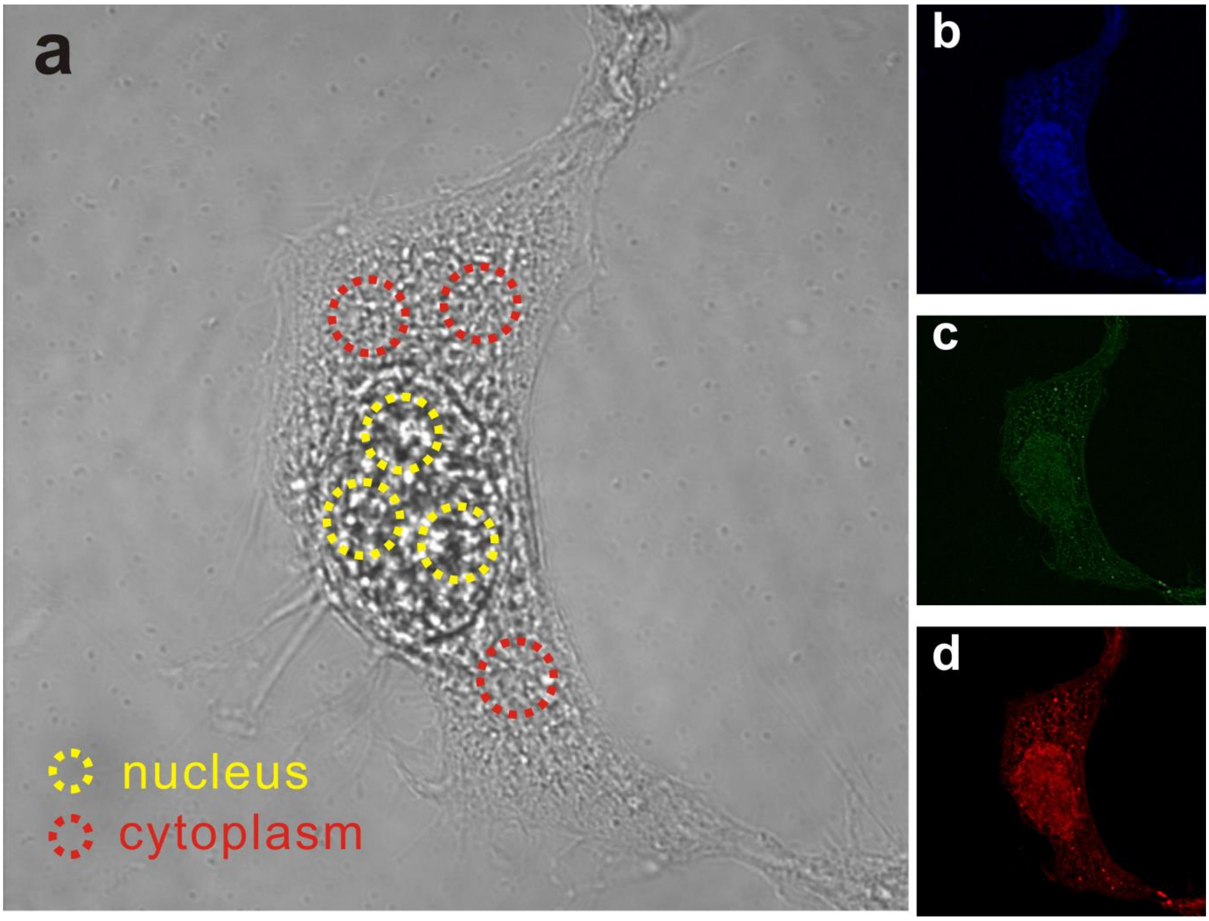

Figure S9. Quantitative analysis of CLSM images taken from HeLa cells incubated with CDs (500 μg/mL) for 24 h. (a) bright-field and under (b) 405 nm, (c) 473 nm, and (d) 559 nm laser excitation.

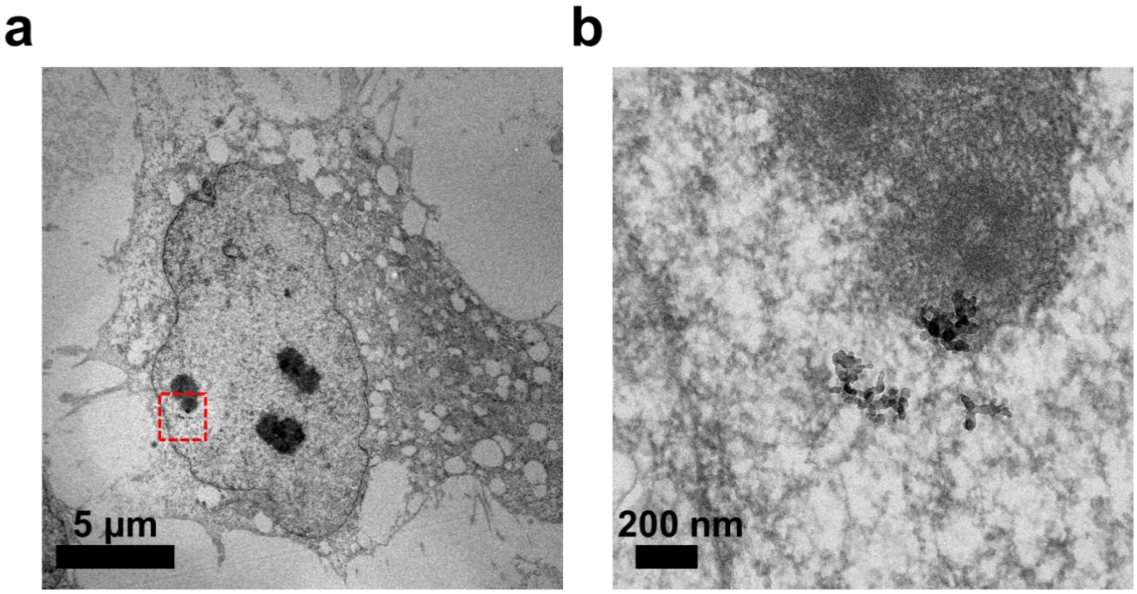

Figure S10. Bio-TEM image of HeLa cells displaying nuclear localization of CDs at different magnification (a) 3,800 × and (b) 26,000×. (b) is a zoom-in image of the red box in (a) image.

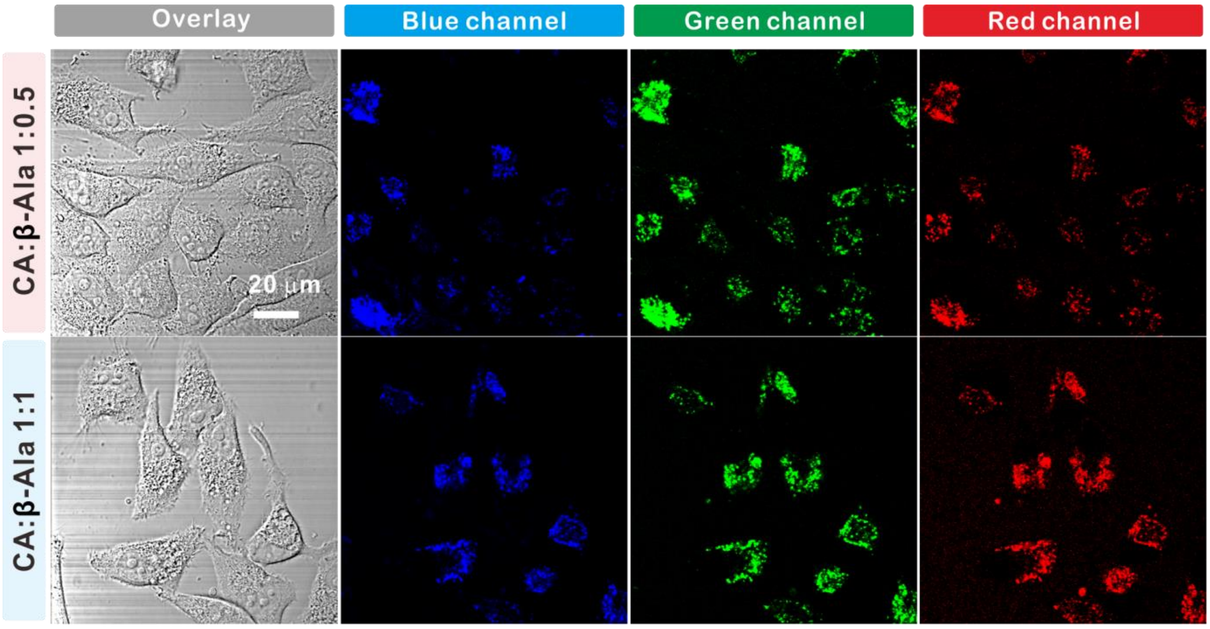

Figure S11. Confocal fluorescence microscopy images showing the cytoplasmic transport of CDs composed of 1:0.5 and 1:1 molar ratio of CA: β-Ala in HeLa cells.

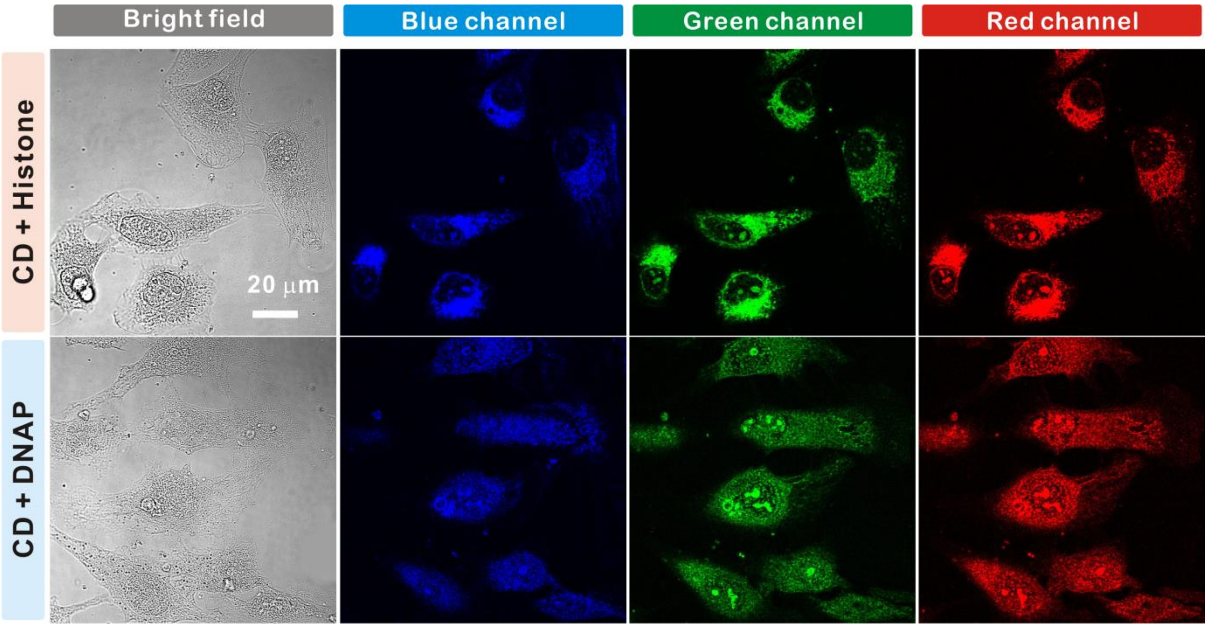

Figure S12. Bright-field and confocal fluorescence images of HeLa cells treated with CD (500 μg/mL) and histone H2A (5 μg) or DNA polymerase (25 U) for 24 h. The CDs co-incubated with histones shows their fluorescence in the cytosol, whereas, the CDs incubated with DNA polymerase are observed in both the cytosol and the nucleus.

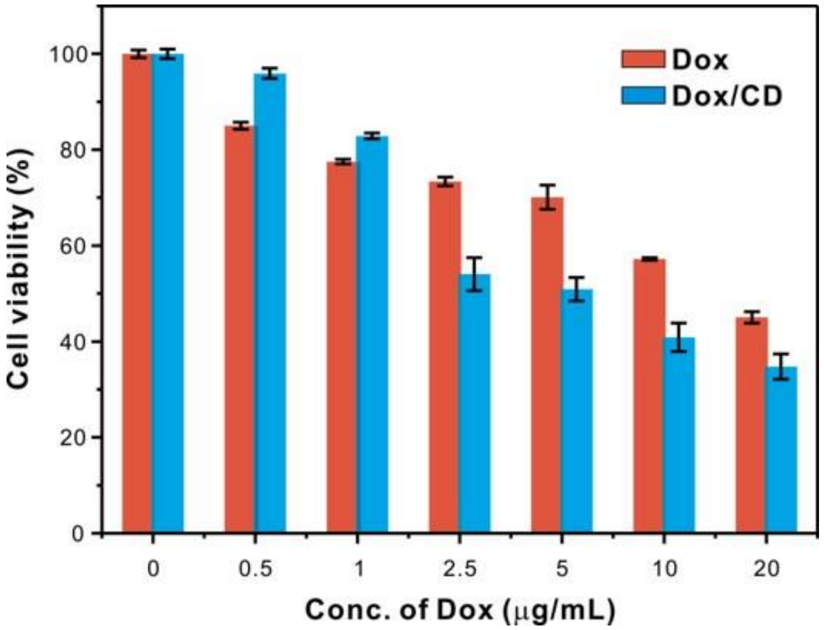

Figure S13. Cell viability of the WI-38 normal cells treated with different concentrations (μg/mL) of Dox alone and Dox/CD for 24 h.
